# Would ivermectin for malaria control be beneficial in onchocerciasis-endemic regions?

**DOI:** 10.1186/s40249-019-0588-7

**Published:** 2019-08-23

**Authors:** Joseph Nelson Siewe Fodjo, Marina Kugler, An Hotterbeekx, Adam Hendy, Jean-Pierre Van Geertruyden, Robert Colebunders

**Affiliations:** 1 Global Health Institute, University of Antwerp, Kinsbergen centrum, Doornstraat 331, 2610 Antwerp, Belgium; 2 Department of Pathology, University of Texas Medical Branch, Galveston, USA

**Keywords:** Malaria, Ivermectin, Onchocerciasis, Mass drug administration, Vector control

## Abstract

**Background:**

There is accumulating evidence supporting the use of ivermectin as a malaria control tool. Recent findings from the repeat ivermectin mass drug administrations for control of malaria trial demonstrated a reduced incidence of malaria in villages which received repeated ivermectin mass drug administration (MDA; six doses) compared to those who had only one round of ivermectin. Several other studies investigating the benefits of ivermectin for malaria purposes are ongoing/planned.

**Main text:**

While ivermectin MDA offers promising perspectives in the fight against malaria, we highlight the added benefits and anticipated challenges of conducting future studies in onchocerciasis-endemic regions, which are confronted with a substantial disease burden including onchocerciasis-associated epilepsy. Increasing the frequency of ivermectin MDA in such places may reduce the burden of both malaria and onchocerciasis, and allow for more entomological investigations on both the *Anopheles* mosquitoes and the blackflies. Upfront, acceptability and feasibility studies are needed to assess the endorsement by the local populations, as well as the programmatic feasibility of implementing ivermectin MDA several times a year.

**Conclusions:**

Onchocerciasis-endemic sites would doubly benefit from ivermectin MDA interventions, as these will alleviate onchocerciasis-associated morbidity and mortality, while potentially curbing malaria transmission. Involving onchocerciasis programs and other relevant stakeholders in the malaria/ivermectin research agenda would foster the implementation of pluri-annual MDA in target communities.

**Electronic supplementary material:**

The online version of this article (10.1186/s40249-019-0588-7) contains supplementary material, which is available to authorized users.

## Multilingual abstracts

Please see Additional file 1 for translations of the abstract into the five official working languages of the United Nations.

## Background

The use of avermectins for vector control has been considered for decades [1]. *Anopheles* mosquitoes were shown to have a reduced life expectancy after biting humans who have ivermectin in their blood [2]. Previous experiments with chickens, pigs and cattle have also demonstrated that ivermectin increases mosquito mortality [3–5]. Although research is yet to ascertain which specific component in ivermectin is responsible for its mosquitocidal effects [6], mass drug administration (MDA) of ivermectin as an endectocide has been proposed as an additional tool to reduce malaria transmission [7]. Of note, the deleterious effects of ivermectin are not limited to the mosquito vectors only, but also extend to the malaria parasite itself [8]. Modelling techniques as well as proof-of-concept field studies have shown that treatment with ivermectin in combination with short courses of currently used artemisinin combination MDA, have the potential to reduce malaria transmission [9, 10]. Safer alternatives such as an ultra-long-acting oral ivermectin formulation [11] and ivermectin-based toxic sugar baits [12] equally offer promising perspectives for future malaria control initiatives. The anti-parasitic drug moxidectin is under consideration for large scale use against onchocerciasis; but in contrast to ivermectin, moxidectin is unable to knockdown the malaria vector after being ingested during a blood meal [13].

In a recent cluster-randomized study (the repeat ivermectin mass drug administrations for control of malaria [RIMDAMAL] trial) performed in a high malaria transmission area in Burkina Faso [10], Foy et al. randomized villages to either an intervention or a control group. All eligible village residents received a single oral dose of ivermectin (150–200 μg/kg) and albendazole (400 mg), while the intervention villages received five additional doses of ivermectin alone at 3-week intervals over an 18-week period. The crude cumulative malaria incidence was lower in the intervention group (648 episodes among 327 children; 2.00 episodes per child) than in the control group (647 episodes among 263 children; 2.49 episodes per child; *P* < 0.0001). After adjusting for sex and village/household clustering, children in the intervention villages had a 20% decreased risk of malaria compared to those in the control group (risk ratio: 0.80 [95% *CI*: 0.70 to 0.91]; risk difference: -0.49 [95% *CI*: − 0.79 to − 0.21], *P* = 0.0009). While these findings clearly demonstrate the benefits of more frequent ivermectin MDA, other similar trials are needed to confirm them.

## Main text

The RIMDAMAL trial adds to existing evidence suggesting that ivermectin may become a new tool to improve malaria control. Moreover, the trial showed that repeated ivermectin MDA seems safe [10]. Presently, ivermectin is commonly used for the control and treatment of onchocerciasis, a filarial disease caused by the nematode *Onchocerca volvulus*, which is transmitted by blackflies (Simuliidae). As an endectocide, ivermectin targets ligand-gated chloride channels in invertebrates, which are necessary for their neuromuscular transmission. While ivermectin is very active against *O. volvulus* microfilariae*,* studies have failed to show an increased mortality among blackflies which fed on ivermectin-treated persons [14]. However, the ivermectin dosage and frequency of administration in the previous studies were lower than the RIMDAMAL regimen.

It would therefore be useful to conduct similar RIMDAMAL trials in places with ongoing onchocerciasis transmission, more so because in several endemic settings is Africa, onchocerciasis is still associated with a substantial burden of disease including onchocerciasis-associated epilepsy (OAE) [15]. By implementing ivermectin MDA several times a year, not only malaria but also onchocerciasis-associated morbidity and mortality can be prevented. During such trials in onchocerciasis foci, entomological investigations should be conducted concomitantly to investigate the effect of different ivermectin regimens on *Anopheles*/blackfly survival. Blood obtained from trial participants can be ingested by non-infected *Anopheles*/blackflies from an insectary using membrane-feeding methods in a laboratory setting, followed by close monitoring of the insects. Indeed, the membrane-feeding approach is reliable and more ethically acceptable than exposing human baits to natural insects’ bites. Another advantage of conducting such research in onchocerciasis-endemic regions is that there are already existing networks of community-directed distributors of ivermectin, with field experience in MDA.

Currently, onchocerciasis transmission is still ongoing in several endemic sites due to suboptimal adherence to yearly community-directed treatment with ivermectin (CDTI) [15]. Consequently, we expect that implementing pluri-annual ivermectin MDA and achieving a satisfactory coverage in these communities would entail a number of constraints including logistical issues, financial limitations, and increased risk of adverse events (Table1). Therefore, while new trials to investigate the strategy of repeated ivermectin treatments to reduce malaria transmission are being planned, feasibility studies should be undertaken using mixed methods. The acceptability, potential endorsement by the local population, programmatic feasibility, and the cost of implementing ivermectin MDA several times a year should be assessed before next big studies are conducted. Additionally, the sustainability of such a scheme would require greater quantities of ivermectin and more local resources for its distribution; this can only be achieved via an increased commitment of pharmaceutical companies and availability of multilateral funds to finance drug access in the long term [7].

**Table 1:** Possible challenges to implement multiple ivermectin treatments annually

Challenge	Possible explanation	Remarks	Proposed perspectives
Increased cost	- Reticence from drug manufacturers to provide additional huge quantities of ivermectin for free. - Increased local cost for ivermectin distribution, because of more logistical needs and multiplied labour force.	Multiple CDTI per year would increase absolute cost, but the public health benefits for both malaria and neglected tropical diseases would be huge.	- Secure external funding to sustain pluri-annual CDTI. - Convince drug manufacturers to continue providing the drug for free.
Reduced compliance	- Fear of side effects. - Ignorance, lack of education and sensitization about CDTI.	Most adverse effects of ivermectin are due to an initially high microfilarial density or loasis co-infection. Rarely, adverse effects may still occur outside these circumstances but often requires abnormally high drug dosages and/or frequency of administration.	- Use a test-and-not-treat approach in co-endemic settings [16]. - Respect dosages of 150–200 μg/kg. - Educate the population on the health benefits of CDTI. - Reassure the population that side effects will decrease with subsequent doses of ivermectin, as the microfilarial density keeps reducing.
Drug resistance	Increased exposure to ivermectin due to a higher frequency of treatment may induce ivermectin resistance in the parasite [17].	Polytherapy could help prevent drug resistance to a single drug. During the RIMDAMAL study for instance, ivermectin was co-administered with albendazole.	- Monitor drug resistance. - Provide ivermectin in combination with other drugs using an integrated MDA approach [18].
Drug interactions	Ivermectin can interact with a number of molecules, including anti-infectious agents [19].	Important for drugs that are taken regularly, such as anti-epileptic drugs or antiretroviral drugs.	Individuals presenting a risk for drug interactions should not receive a multi-dose ivermectin treatment regimen.

*CDTI* Community-directed treatment with ivermectin*, MDA* Mass drug administration*, RIMDAMAL* Repeat ivermectin mass drug administrations for control of malaria

An intriguing finding of the RIMDAMAL trial that is of interest for MDA programs, is that a relatively large number of under-five children were treated with ivermectin and no drug-related side effects were observed [10]. These results suggest that the frequent exclusion of children aged 5 years and below during CDTI in many endemic communities needs to be reconsidered. This is important because untreated children who are infected with *O. volvulus* constitute a human reservoir for the parasite. Also, in settings of high onchocerciasis transmission, OAE including nodding syndrome commonly occurs in children as young as 3 years [15]. Moreover, given that the risk of developing OAE depends on the intensity of *O. volvulus* infection during childhood [20], it is possible that OAE will altogether be averted in children who receive ivermectin early enough as this may prevent a peak microfilarial density capable of triggering seizures. Therefore, clinical trials investigating the safety of ivermectin in children below 5 years and/or 15 kg of body weight should be considered.

Finally, Foy et al. [10] conducted the RIMDAMAL study only during the rainy season as they expected a higher malaria incidence during that period. Their observations showed that the difference in malaria incidence between the two study groups was widest 1–2 weeks after the third and sixth ivermectin MDA. It is unlikely that the observed pattern is related to rainfall levels during the study, as the authors themselves attributed it to the pharmacokinetics and mosquitocidal activity of ivermectin [10]. Ideally, we suggest a study in which ivermectin MDA would be initiated 1–2 weeks before the rainy season, such that the ivermectin concentration in treated participants will be sufficiently high by the time rainfall resumes. We realise however that we have limited ability to predict rains and that, given the short half-life of ivermectin, such an approach may require repeated/multiple doses of ivermectin in the event of delayed rainfalls.

## Conclusions

As the research agenda concerning the use of ivermectin for malaria control is advancing [7], it would be beneficial to collaborate with onchocerciasis control programs to discuss eventual possibilities of increasing ivermectin MDA frequency in areas which are co-endemic for both onchocerciasis and malaria. Potentially, this may reduce malaria transmission, but it will certainly reduce onchocerciasis-associated morbidity and mortality.

## Additional file

Additional file 1: Multilingual abstracts in the five official working languages of the United Nations. (PDF 488 kb)

## Data Availability

Not applicable.

## Abbreviations

CDTI: Community-directed treatment with ivermectin
MDA: Mass drug administration
OAE: Onchocerciasis-associated epilepsy
RIMDAMAL: Repeat ivermectin mass drug administrations for control of malaria
