# Supplementary material for: Would ivermectin for malaria control be beneficial in onchocerciasis-endemic regions?
Source: Infect Dis Poverty. 2019 Aug 23;8:77. doi: 10.1186/s40249-019-0588-7 (PMC6706915; doi:10.1186/s40249-019-0588-7)
Supplement: Supplementary file 1 — Multilingual abstracts in the five official working languages of the United Nations. (PDF 488 kb) [file 40249_2019_588_MOESM1_ESM.pdf]

Translation of the abstract into the five official working languages of the United Nations

هل سيفيد الإيفرمكتين في مكافحة الملاريا في المناطق الموبوءة بداء العمى النهري؟

جوزيف نيلسون سيو فودجو، مارينا كوغلر، آن هوتربيكس، آدم هندي، جان بيير فان جيبرترودن جان بيير، روبرت كوليبوندرز

ملخص
معلومات أساسية: هناك أدلة متزايدة تدعم فرضية استعمال الإفرمكتين كدواء للتحكم في الملاريا. أظهرت آخر النتائج المستخلصة من تجربة إعطاء دواء الإيفرمكتين بشكل متكرر للسيطرة على الملاريا انخفاضًا في معدل الإصابة به في القرى التي تلقت عدة جرعات من الإيفرمكتين (MDA ؛ ست جرعات) مقارنة بأولئك الذين تناولوا جرعة واحدة فقط من الإيفرمكتين. العديد من الدراسات الأخرى التي تبحث عن فوائد الإيفرمكتين في محاربة الملاريا جارية أو تم التخطيط لها.
النص الرئيسي:على الرغم من أن هناك بوادر واعدة للإيفرمكتين (MDA) في مكافحة الملاريا ، لكننا نسلط الضوء على الفوائد المضافة والتحديات المتوقعة لإجراء دراسات مستقبلية في المناطق الموبوءة بمرض العمى النهري، والتي تواجه عبء داء كبير بما في ذلك الصرع المصاحب لمرض العمى النهري. إن زيادة وتيرة استعمال عقار الإيفرمكتين (MDA) في مثل هذه المناطق قد يقلل من وزر كل من الملاريا وداء العمى النهري، ويسمح بإجراء مزيد من الدراسات المتعلقة بالحشرات على كل من بعوض الملاريا والذباب الأسود. هناك حاجة إلى إجراء دراسات جدوى ومقبولية مسبقًا لتقييم تأييد السكان المحليين، فضلاً عن عمل جدوى برنامجية لتطبيق الإيفرمكتين(MDA) عدة مرات في السنة.
الاستنتاجات: بإمكان المناطق التي تفشى فيها داء العمى النهري أن تستفيد بشكل مضاعف من إجراءات تدخل دواء الإيفرمكتين (DAM)، حيث إنها ستخفف من نسبة انتشار المرض والوفيات الناجمة عنه، في الوقت الذي من الممكن أن تقوم فيه بالحد من انتقال الملاريا. إن إشراك المعنيين بالأمر ببرامج داء العمى النهري وغيرهم في أجندة أبحاث الملاريا وكذا الإيفرمكتين من شأنه أن يعزز تطبيق (DAM) لعدة سنوات في المجتمعات المستهدفة.

Translated from English version into Arabic by Riyad Zouag, proofread by Samah Almoghrabi, through

TRANSLATORS
WITHOUT BORDERS

伊维菌素是否有助于盘尾丝虫病流行地区的疟疾控制？

Joseph Nelson Siewe Fodjo, Marina Kugler, An Hotterbeekx, Adam Hendy, Jean-Pierre Van geertruyden Jean Pierre, Robert Colebunders

摘要
引言：越来越多的证据支持使用伊维菌素作为疟疾防治工具。最近，重复使用伊维菌素进行群体化疗防治疟疾试验表明，与仅接受一轮伊维菌素 MDA 治疗的村庄相比，接受 6 次伊维菌素 MDA 的村庄疟疾发病率较低。目前，正在进行/计划进行另外几项研究也在探索伊维菌素治疗疟疾的益处。
主要内容：尽管伊维菌素 MDA 在抗击疟疾方面具有一定的前景，但我们需要注意在盘尾丝虫病流行地区开展未来研究的额外益处和预期挑战，这些地区面临着严重的疾病负担，包括盘尾丝虫病相关性癫痫。在这些地方增加伊维菌素 MDA 的频率可能会减轻疟疾和盘尾丝虫病的负担，并有机会对按蚊和黑蝇进行更多的昆虫学研究。但是首先需要进行可接受性和可行性研究，以评估当地居民的认可情况，以及每年实施多次伊维菌素 MDA 的方案可行性。

结论：盘尾丝虫病流行地区将从伊维菌素 MDA 干预措施中获得双重好处，因为这些干预措施将减轻盘尾丝虫病相关的发病率和死亡率，同时可能抑制疟疾的传播。将盘尾丝虫病项目和其他相关利益攸关方纳入疟疾/伊维菌素研究议程将有助于在目标社区实施多年度 MDA。

Translated from English version into Chinese by Xin-Yu Feng, edited by Pin Yang

L'ivermectine comme outil de lutte contre le paludisme serait-elle bénéfique dans les régions d'endémie de l'onchocercose?

Joseph Nelson Siewe Fodjo, Marina Kugler, An Hotterbeekx, Adam Hendy, Jean-Pierre Van Geertruyden, Jean Pierre, Robert Colebunders

Résumé

Contexte: De plus en plus de preuves militent en faveur de l'utilisation de l'ivermectine comme outil de lutte contre le paludisme. Des résultats récents issus de l'essai « repeat ivermectin mass drug administrations for control of malaria » (administrations massives répétées de médicaments à l'ivermectine dans le cadre de la lutte contre le paludisme) ont montré une incidence réduite du paludisme dans les villages ayant reçu une administration massive de médicaments (AMM; six doses) répétée à l'ivermectine par rapport à ceux n'ayant reçu qu'un seul cycle d'ivermectine. Plusieurs autres études sur les avantages de l'ivermectine dans le cadre de la lutte contre le paludisme sont en cours / prévues.

Texte principal: Alors que l'AMM à l'ivermectine offre des perspectives prometteuses dans la lutte contre le paludisme, nous mettons en avant les avantages supplémentaires et les difficultés anticipées de la réalisation d'études futures dans les régions d'endémie de l'onchocercose, confrontées à une charge de morbidité importante, notamment imputable à l'épilepsie associée à l'onchocercose. L'augmentation de la fréquence de l'AMM à l'ivermectine dans ces régions peut réduire la charge du paludisme et de l'onchocercose et permettre davantage d'enquêtes entomologiques sur les moustiques *Anopheles* et les simulies. Des études préalables, d'acceptabilité et de faisabilité sont nécessaires pour évaluer l'approbation par les populations locales, ainsi que la faisabilité programmatique de la mise en œuvre de l'AMM à l'ivermectine plusieurs fois par an.

Conclusions: Les sites d'endémie de l'onchocercose tireraient un double bénéfice des interventions d'AMM à l'ivermectine, dans la mesure où elles permettraient de réduire la morbidité et la mortalité associées à l'onchocercose, tout en réduisant potentiellement le risque de transmission du paludisme. L'implication des programmes de lutte contre l'onchocercose et des autres parties prenantes concernées dans le programme de recherche sur le paludisme / l'ivermectine favoriserait la mise en œuvre de l'AMM pluriannuelle dans les communautés cibles.

Translated from English version into French by Sarah Merlin, proofread by Maëva Paoletti, through

Будет ли лечение ивермектином эффективным в регионах, эндемичных по онхоцеркозу?

Джозеф Нельсон Сиве Фоджо (Joseph Nelson Siewe Fodjo), Марина Куглер (Marina Kugler), Ан Хоттербикс (An Hotterbeekx), Адам Хенди (Adam Hendy), Жан-Пьер ван Геертруйден Жан Пьер (Jean-Pierre Van geertruyden Jean Pierre), Роберт Коулбандерс (Robert Colebunders)

Аннотация

Контекст исследования. Имеется все больше доказательств, подтверждающих эффективность использования ивермектина в качестве инструмента контроля малярии. Недавние результаты повторного исследования по многократному введению ивермектина для контроля за развитием малярии свидетельствуют о снижении малярии в сельской местности, где пациентам повторно многократно вводили ивермектин (MDA; шесть доз) по сравнению с теми, кто получил только один курс лечения ивермектином. Планируются или проводятся несколько других исследований, в которых изучается польза ивермектина в целях лечения малярии.

Основной текст. В то время как при массовом введении ивермектина открываются многообещающие перспективы в борьбе против малярии, мы подчеркнем дополнительные преимущества и текущие проблемы проведения последующих исследований в регионах, эндемичных по онхоцеркозу, что сопоставляется со значительным бременем заболевания, включая эпилепсию, связанную с онхоцеркозом. Увеличение частоты многократного введения ивермектина в такой местности может уменьшить бремя и малярии, и онхоцеркоза, и способствовать большему числу энтомологических исследований как москитов вида *Anopheles*, так и мошек. Первоначально необходимо провести исследования по приемлемости и осуществимости для оценки одобрения местным населением, а также программной осуществимостью внедрения массового введения ивермектина несколько раз в год.

Выводы. Проведение мероприятий по массовому введению ивермектина будет благоприятно влиять на районы, эндемичные по онхоцеркозу, т. к. это уменьшит заболеваемость, связанную с онхоцеркозом и смертность, и возможно сдержит передачу малярии. Включение программ по лечению онхоцеркоза и других соответствующих субъектов в повестку исследований малярии/ивермектина будет способствовать внедрению многолетнего многократного введения лекарств в целевых сообществах.

Translated from English version into Russian by Veronika Demeshchyk, proofread by Michael Orlov, through

TRANSLATORS
WITHOUT BORDERS

¿Será beneficioso ivermectina, para el control de la malaria, en regiones endémicas con oncocercosis?

Joseph Nelson Siewe Fodjo, Marina Kugler, An Hotterbeekx, Adam Hendy, Jean-Pierre Van geertruyden Jean Pierre, Robert Colebunders

Resumen

Antecedentes: Existe una evidencia acumulativa, que apoya el uso de ivermectina, como herramienta para el control de la malaria. Los descubrimientos recientes, partiendo de las repetidas administraciones de medicamentos masivos con ivermectina, para el control de la malaria, demostraron una menor incidencia de malaria en las aldeas que recibieron una administración masiva repetida con ivermectina (MDA; seis dosis), en comparación con aquellos que solo recibieron una ronda de ivermectina. Varios otros estudios, que investigan los beneficios de la ivermectina para la malaria, están en curso o se están plananificando.

Texto principal: Si bien la ivermectina MDA ofrece perspectivas prometedoras, en la lucha contra la malaria, destacamos beneficios adicionales y desafíos anticipados en la realización de futuros estudios, en regiones endémicas de oncocercosis, las cuales se enfrentan a una carga de enfermedad sustancial, entre la que se incluye la epilepsia asociada a oncocercosis. El aumento de la frecuencia de ivermectina MDA, en dichos lugares, puede reducir la carga de la malaria y la oncocercosis, y permite más investigaciones entomológicas tanto en los *mosquitos y moscas negras* Anopheles. Se necesitan estudios iniciales de aceptabilidad y viabilidad, para evaluar el respaldo de las poblaciones locales, así como la viabilidad programática de implementar ivermectina MDA, varias veces al año.

Conclusiones: Los lugares endémicos de oncocercosis se beneficiarán doblemente de las intervenciones de ivermectina MDA, ya que estas aliviarán la morbilidad y mortalidad asociadas con la oncocercosis, al tiempo que podrán frenar la transmisión de la malaria. La participación de los programas de oncocercosis y otros actores interesados pertinentes, en el programa de investigación sobre la malaria/ivermectina, fomentará la aplicación de la MDA multianual, en las comunidades destinatarias.

Translated from English version into Spanish by Ilduara Escobedo, proofread by María Luz Puerta, through

TRANSLATORS
WITHOUT BORDERS
